# *Ricinodendron heudelotii* (Euphorbiaceae) seed oil prevents DMBA-induced breast cancer under menopause-like conditions in Wistar rats

**DOI:** 10.3389/fphar.2024.1389976

**Published:** 2024-05-16

**Authors:** Stéphane Zingué, Edwige Nana Tchoupang, Linda Takou Madji, Boris Hugor Pehuie Fomat, Borelle Mafogang, Dieudonné Njamen, Joseph Marie Nkodo Mendimi

**Affiliations:** ^1^ Department of Pharmacotoxicology and Pharmacokinetics, Faculty of Medicine and Biomedical Sciences, University of Yaounde 1, Yaounde, Cameroon; ^2^ Department of Medical and Biomedical Engineering, Higher Technical Teachers’ Training College, University of Ebolowa, Ebolowa, Cameroon; ^3^ Department of Animal Science, Faculty of Agriculture and Veterinary Medicine, University of Buea, Buea, Cameroon; ^4^ Department of Animal Biology and Physiology, Faculty of Science, University of Yaoundé 1, Yaounde, Cameroon; ^5^ Department of Morphological Sciences and Pathological Anatomy, Faculty of Medicine and Biomedical Sciences, University of Yaounde 1, Yaounde, Cameroon

**Keywords:** *Ricinodendron heudelotii*, breast cancer, inflammatory cytokines, DMBA, ovariectomy

## Abstract

Despite efforts, breast cancer remains associated with a high incidence and mortality rate. *Ricinodendron heudelotii* also known as “Njansang,” is a plant used for cancer treatment. While several reports on the anticancer potential of its leaves exist, little is known about its seed oil. This study aimed to evaluate the *in vitro* and *in vivo* anti-breast cancer activity of “Njansang” seed oil. The inhibitory effect of “Njansang” seed oil was determined using MTT and CCK-8 dye reduction assays. Breast cancer was induced with DMBA and promoted with E_2_V (1 mg/kg) for 4 weeks in ovariectomized rats (menopausal condition). Evaluated parameters included tumor incidence, tumor mass and volume, histopathology, breast cancer biomarker CA 15–3, antioxidant status (CAT, GSH, MDA, NO, SOD), TNF-α and INFγ levels, lipid profile (total cholesterol, LDL-cholesterol, triglycerides and HDL-cholesterol), as well as toxicity parameters (ALT, AST, creatinine). “Njansang” oil significantly reduced the growth of ER+ (MCF-7) and triple negative (MDA-MB 231) adenocarcinoma cells *in vitro* as well as tumor incidence, tumor mass and CA 15–3 levels *in vivo*. It exhibited antioxidant activity, characterized by an increase in SOD and catalase activities, GSH levels and decreased MDA levels compared to the DMBA group. TNF-α and INF-γ levels were reduced following oil treatment, while total cholesterol, LDL-cholesterol and triglyceride levels were reduced. The aforementioned findings confirm the protective effects of “Njansang” oil on induced breast cancer in ovariectomized rats.

## 1 Introduction

Health who is defined as “a state of complete physical, mental and social wellbeing and not merely the absence of disease or infirmity” requires the fulfillment of an individual’s basic needs, such as emotional, health, nutritional, social and cultural needs, throughout life (WHO, 1946). In recent years, there has been considerable attention given to health issues, particularly breast cancer. Cancer, a multifactorial genetic disease, involves uncontrolled division and proliferation of abnormal cells that can spread to other tissues (Akhouri et al., 2020). Sung et al. (2021) reported an estimated −19.3 million new cancer cases with over 10 million deaths by 2020 (Globocan, 2020). Without intervention, it is projected that the number of new cancer cases worldwide will increase by 49%, with deaths rising by 62% by 2040 (Cao et al., 2021). Despite becoming the most diagnosed cancer globally in 2020, breast cancer remains one of the most significant health problems among women worldwide (Sharma, 2019). In Africa, it stands as the leading cause of death in women, accounting for 28% of all cancers and 20% of all cancer deaths in women (Ferlay et al., 2020). In Cameroon, it remains a major public health concern affecting women, with over 4170 (20.1%) new cases and 2108 (16%) deaths per year (Globocan, 2020; Zingue et al., 2021). The etiology of breast cancer includes, but is not limited to, age, genetic factors (mutations in the BRCA1 and BRCA2 suppressor genes), prolonged exposure of the breast to endogenous and exogenous estrogens (Sun et al., 2017). Additionally, environmental factors such as lifestyle changes, cigarette smoke, grilled and smoked meat and environmental pollutants, notably polycyclic aromatic hydrocarbons (PAHs, e.g., DMBA), increase the risk of developing breast cancer.

Drugs used to treat this deadly disease include tamoxifen (an anti-estrogen hormone therapy), doxorubicin and paclitaxel (chemotherapy drugs) (Chaurasia et al., 2023). However, limitations have been associated with the use of these anticancer drugs including their high cost and long-term adverse effects (cardio-toxicity and endometrial cancer) (Tommasi et al., 2022). Alternatively, natural substances have been used to combat cancers, particularly in developing countries where 80% of individuals resort to herbal remedies. This preference is due to the wide range of biological activities, affordability and minimal side effects associated with these medications (Khazaei et al., 2015). Although many patients opt for traditional alternatives, they often express dissatisfaction with the prolonged treatment duration and laborious preparation processes of herbal remedies. Consequently, there is an increasing appreciation for alicaments valued for their dual function as both nutrient and medicine, with one such substance being *Ricinodendron heudelotii*.

*Ricinodendron heudelotii* is a tree that can reach 30 m in height with a circumference of 3 m (Noumi, 1984). It grows in the equatorial forest ecosystems of Madagascar and the west coast of Africa. It is used in traditional medicine to treat various ailments, including cancer. The leaves have been used to treat dysentery (Momeni et al., 2010). Consumption of *R. heudelotii* almond oil by male Sprague-Dawley rats showed reduced cholesterol and triglyceride levels compared to controls (Agbor and Naidoo, 2015). *R. heudelotii* oil is rich in polyunsaturated fatty acids, notably including α-eleostearic acid, linoleic acid, β-eleostearic acid, and catalpic acid, which constitute the primary constituents of its triglycerides. The abundance (nearly 50% of total fatty acids) of α-eleostearic acid (C18:3) and its isomers (mainly conjugated) within the oil offers numerous health advantages and holds promise for diverse applications in the pharmaceutical sector (Nikiema et al., 2019). While one study has investigated the effects of conjugated fatty acids on certain cancers, to the best of our knowledge no animal model study has been reported. The aim of this study was to assess the protective effect of “Njansang” oil against induced breast cancer in ovarectomized rats. Specifically, we aimed to evaluate its effects on the incidence, burden, morphology (relative mass and volume) and histology of breast tumors. Additionally, we assessed the breast cancer biomarker (CA15, certain inflammatory cytokines (TNF-α and INF-γ) of interest in breast cancer as well as the oxidative status in the rat mammary gland (SOD, GSH, catalase, NO and MDA) and lipid profile (total cholesterol, HDL, LDL cholesterol and triglyceride).

## 2 Materials and methods

### 2.1 Substances

7,12-dimethylbenz(a)anthracene (DMBA) was obtained from Sigma-Aldrich (Starnberg, Germany) and tamoxifen citrate (Mylan^®^) was purchased from MYLAN SAS (Saint-Priest, France). The anesthetics diazepam (Valium^®^ 10 mg/2 mL) and ketamine (Ketamine hydrochloride^®^ 50 mg/mL) were obtained from Roche (Fontenay-sous-bois, France) and Rotex Medica (Trittau, Germany) respectively. 17β-estradiol Valerate (E_2_V) (Progynova^®^ 2 mg) was obtained from Delpharm (Lille, France) and genistein was obtained from “Extrasynthese^®^” (Genay, France).

### 2.2 Harvest, identification and oil extraction of *Ricinodendron heudelotii*

The leaves, twigs and seeds of *R. heudelotii* (Euphorbiaceae) were harvested in Bandjoun (West Region, Cameroon) and authenticated by comparison with a sample deposited in the National Herbarium of Cameroon (voucher N° 50811/NHC). Subsequently, the “Njansang” seeds underwent about 2 weeks of fermentation to promote pulp rot as previously reported (Dupriez and De Leener, 1987; Tiki Manga et al., 2000). Then, the pulped seeds were boiled in water for 2 h to weaken the sclerotic shell and left overnight. The kernels obtained by opening the shell were then shade-dried for a week. Following drying, solvent-free cold extraction was performed using a cold oil extraction machine.

### 2.3 *In vitro* anticancer investigation

#### 2.3.1 Cell culture

Human estrogen non-sensitive (MDA-MB 231) and sensitive (MCF-7) cell lines were supplied by American Type Culture Collection (ATCC) Promochem (Wesel, Germany). Cells were grown and subcultured in RPMI-1640 media supplemented with 10% fetal bovine serum (FBS), and 1% penicillin (100 U/mL)/streptomycin (100 g/mL). They were incubated in a humid 5% CO_2_ incubator maintained at 37°C and 7.4 pH, with 90% of the supernatant replaced with fresh medium every 2 days during cell passage.

#### 2.3.2 Assessment of cell growth

Cell growth was assessed using the mitochondrial tetrazolium test (MTT) (Roche Diagnostics, Penzberg, Germany). First, the number of viable cells was estimated using the enzyme-linked immunosorbent assay (ELISA) Multiskan TECAN reader counter system. Subsequently, 100 μL of cells (1 × 10^5^ cells/mL) were seeded onto 96-well plates. *R. heudelotii* oil was tested at final concentrations of 2.5–200 μg/mL. Control cells were exposed to vehicle (DMSO 0.01%) only. At 0, 24, 48, and 72 h, 10 μL of MTT (5 mg/mL) was added and incubated at 37°C, 5% CO_2_ for 2 h; afterward the cells were lysed in a buffer containing 10% SDS in 0.01 M HCl for 2 additional hours. Absorbance at 550 nm was determined for each well using a microplate ELISA TECAN^©^ SPARK reader (Crailsheim, Germany).

#### 2.3.3 Assessment of cell proliferation

The stable and non-toxic GLPBio Cell Counting Kit-8 (CCK-8) from Hamburg, Germany was used to estimate the ability of *R. heudelotii* oil to inhibit breast cancer cells *in vitro*. Cells (100 μL, 1 × 10^4^ cells/mL) were seeded onto 96-well plates and incubated (5% CO_2_, 37°C) for 24 h. Thereafter 10 µL of the retained freshly prepared oil was tested at concentrations of 0.5–40 μg/mL, while the control was exposed to vehicle (DMSO 0.01%). After 48 h of incubation, 10 µL of CCK8 solution was added to each well of the plate and incubated for 4 h (5% CO_2_, 37°C). Plates were gently homogenized on a shaker and absorbance was read at 450 nm using a microplate TECAN reader.

### 2.4 *In vivo* anticancer investigation

#### 2.4.1 Experimental animals

For this study, 36 albino female rats of Wistar strains, aged 6–10 weeks (70–120 g), were obtained from the Animal Physiology facility of the University of Yaoundé I. These rats were reared in large plastic cages, with six animals per cage, at room temperature. They had free access to water and a standard diet composed of corn-meal (36.7%), wheat meal (36.6%), bone meal (14.5%), crushed fish (4.8%), palm kernel cake (7.3%), sodium chloride (0.3%) and vitamin (0.01%).

Animal handling and treatments were approved by the Joint Institutional Review Board on Animal & Human Bioethics of the Faculty of Science (University of Yaoundé 1) reference # BTC-JIRB2021-010, which is in line with the European Union on the care of animals (EEC Council 86/609).

#### 2.4.2 Dose determination

The carcinogen DMBA was used to induce mammary tumors at a dose of 50 mg/kg BW dissolved in 1 mL olive oil (normal spleen received olive oil only) (Ramachandran et al., 2017). Tamoxifen (Tamox), a selective estrogen receptor modulator (SERM) was used as a positive control in this study and administered at a dose of 3.3 mg/kg BW (Maltoni et al., 1997). “Njansang” oil was administered at a dose of 1 mL/kg. Estradiol valerate (E_2_V) (Progynova^®^) was administered at a dose of 1 mg/kg BW per route.

#### 2.4.3 Hormone depletion induction protocol: oophorectomy

Female rats were anesthetized with valium (diazepam) and ketamine administered *i.p*. at doses of 10 mg/kg BW and 50 mg/kg BW, respectively. The animals were then placed prone in a dissection tray and a longitudinal opening of approximately 2 cm was made in the back. After opening the peritoneum, the ovaries were exposed and separated from the uterine horn to which they were attached, using scissors. After this operation, the uterine horn was reintroduced into the abdominal cavity and the dorsal opening was closed with sutures.

#### 2.4.4 DMBA-induced breast cancer in ovariectomized rats

To evaluate the protective effect of “Njansang” oil on a DMBA-induced breast cancer model, 42 Wistar rats aged 6–10 weeks (70–120 g) were acclimatized for 17 days, randomized, marked and divided into 7 groups of 6 animals each, then ovariectomized, except for the normal group (sham-operated), and treated for 10 days with amoxicillin and betadine to prevent infection. The normal (NOR, sham-operated rats), ovariectomized (OVX-1, ovariectomized rats) and negative (OVX-2 + DMBA, DMBA-exposed OVX rats) control groups all received distilled water (vehicle). The Tamox + DMBA group, consisting of positive control rats, received tamoxifen at a dose of 3.3 mg/kg body weight. The oil groups, serving as test groups, were comprised of OVX rats treated with “Njansang” oil either 2 times/week (oil 1), 4 times/week (oil 2) or daily (oil 3). All these treatments were administered at different frequencies by intra-gastric gavage. All groups received a single dose (50 mg/kg BW) of a solution of DMBA dissolved in 1 mL olive oil via the subcutaneous intra-mammary route, except for the normal (NOR, sham-operated rats) and ovariectomized (OVX-1, ovariectomized rats) control groups. Five hours after cancer induction, all DMBA-treated rats were treated with amoxicillin at a dose of 89 mg/kg for 10 days via intra-gastric gavage to prevent infection. The breast cancer thus initiated was promoted by administration of estradiol *per os* for 4 weeks. Weight was recorded weekly and the animals were palpated twice a week to detect any mammary tumors. Throughout the experiment, rats were weighed weekly and palpated twice weekly for mammary tumors. After 25 weeks, surviving animals were sacrificed by decapitation under light anesthesia after a 12-h fast.

### 2.5 Organ collection

Blood samples were collected in anticoagulant tubes (ethylene diamine tetra-acetic-EDTA) and dry tubes (centrifuged at 600 *g* for 15 min at 4°C to obtain serum) for evaluation of hematological and biochemical parameters. Additionally, mammary tumors were removed, counted, weighed and measured with a 1 mm precision caliper (IGAGING^®^). Mammary tumors, mammary glands, liver, spleen, lungs, adrenal gland, femur and kidneys were also excised, weighed and immediately fixed in 4% formalin containing 5% NaCl for subsequent histological analysis.

Relative organ weight was expressed in mg/kg BW using the following formula: organ weight (mg/kg) = organ weight (mg)/body weight (kg). Tumor burden (g) = Σ tumor weight (g). % inhibition = (DMBA tumor load—test group tumor load)/tumor load (DMBA). Inhibition % = (Relative mass of tumors (DMBA) - Relative mass (of test groups)/Relative mass (DMBA). It corresponds to the number of animals with at least one tumor, expressed as a percentage of inhibition. Tumor volume (cm^3^) = length × width × thickness × π/6.

### 2.6 Histological analysis

In this study, we referred to the basic techniques which involved fixing, macroscopy, dehydration, embedding, sectioning, staining and mounting the various tissues. Microscopic analysis and size determination of the various epithelia were conducted using a complete set of equipment consisting of an AxiosKop 40 microscope connected to a computer. Images were transferred, edited and analyzed using MRGrab 1.0 and AxioVision 3.1 software, all supplied by ZEISS (Hallbermoos, Germany).

### 2.7 Hematological analysis

The following hematological parameters were determined: white blood cell count (lymphocytes, monocytes, granulocytes), red blood cell count, blood platelets, hematocrit (Htc), hemoglobin (Hb), mean corpuscular volume (MCV), mean corpuscular hemoglobin concentration (MCHC) at the Biochemistry Laboratory, Yaoundé Central Hospital, Cameroon.

### 2.8 Determination of the serum breast cancer tumor marker CA 15–3

Cell Biolabs’ Cancer Antigen 15–3 ELISA kit was used to measure the level of the breast cancer biomarker CA 15–3 in serum, one of the most clinically circulating prognostic biomarkers. The CA 15–3 assay measures excreted or soluble forms of the Mucin-1 protein (MUC-1), a transmembrane protein consisting of 2 subunits forming a stable dimer expressed at the apical plasma membrane of epithelial cells. The kit has a detection sensitivity limit of 4 U/mL. The test was performed according to the manufacturer’s instructions.

### 2.9 Statistical analysis

Results were expressed as mean ± standard error of the mean (SEM) and analyzed using Graph pad prism 5.0. ANOVA (analysis of variance) followed by Dunnett’s *post hoc* test was used to compare all groups to the negative control group (DMBA). The unpaired Student’s t-test was used to compare differences between the negative control group (DMBA) and the normal control group (NOR). The significance level was set at *p <* 0.05.

## 3 Results

### 3.1 Effect of “Njansang” oil on breast cancer cells growth

**FIGURE 1:**
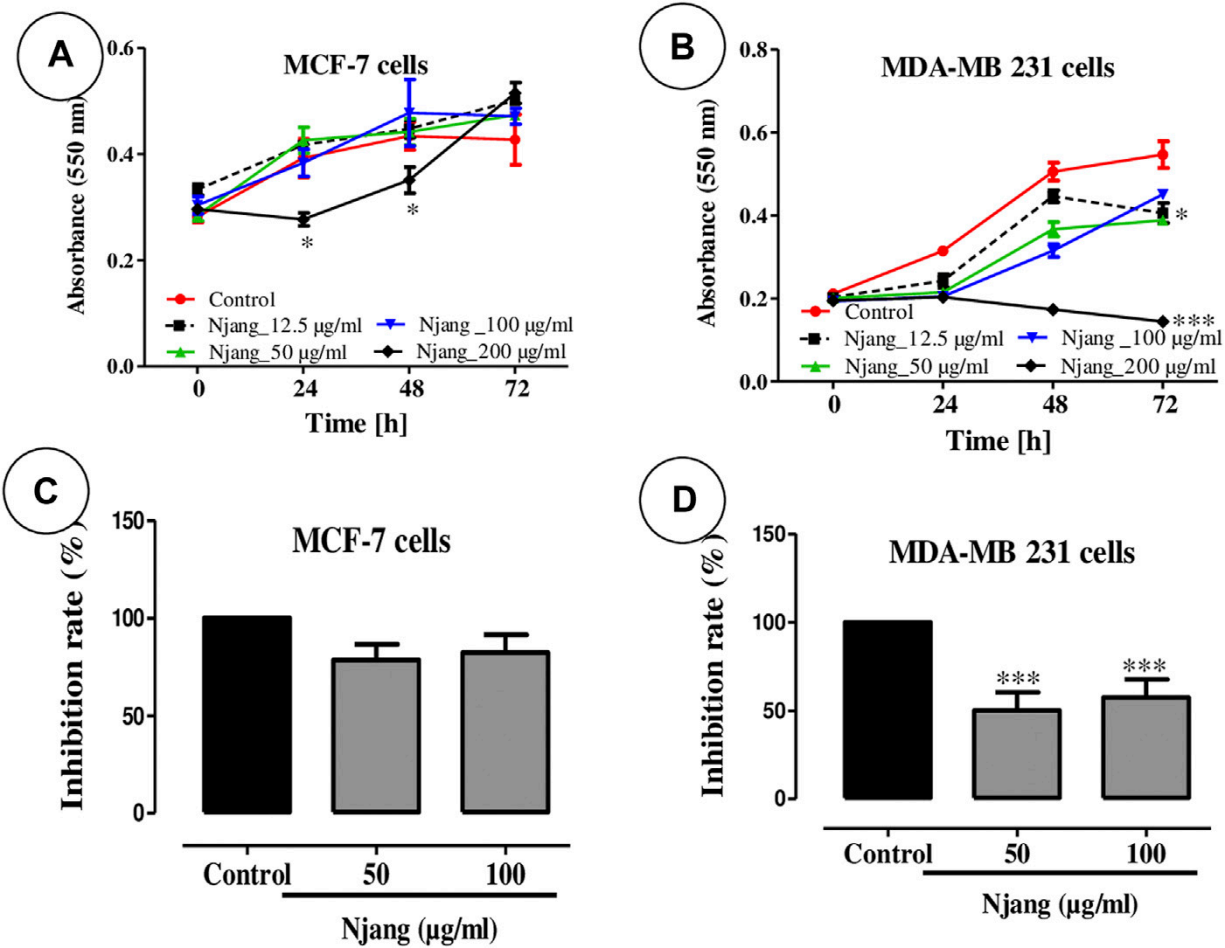
Growth of estrogen-sensitive MCF-7 **(A)** and non-sensitive MDA-MB 231 **(B)** breast adenocarcinoma cells and the proliferation of estrogen-sensitive MCF-7 **(C)** and non-sensitive MDA-MB 231 **(D)** breast adenocarcinoma cells treated with different concentrations of “Njansang” oil after 24, 48, and 72 h. The controls remained untreated. (*n* = 3). Treated cancer cell cultures were compared to non-treated control cultures of the same passage and cell numbers per well. Statistical significance is denoted as *p < 0.05 and ***p < 0.001 compared to control.

Figure 1 depicts the effect of “Njansang” oil on breast cancer cells in culture. MCF-7 cells exhibited less sensitivity compared to MDA-MB 231 cells with a significant (*p <* 0.05) decrease in cell growth observed only at the higher concentration (200 μg/mL) after 24 h and 48 h; however, this effect was lost at 72 h (Figure 1A). Conversely, a concentration-dependent decrease in cell growth was observed in MDA-MB 231 cells significant from 12.5 μg/mL (*p <* 0.05) to 200 μg/mL (*p <* 0.001) concentrations (Figure 1B). IC_50_ was estimated >200 μg/mL in MCF-7 cells while in MDA-MB 231 cells it is around 170 μg/mL after 48 h.

In the same scheme, “Njansang” oil significantly reduced the proliferation rate of MDA-MB 231 cells in culture at 50 and 100 μg/mL (*p <* 0.001), but failed to do so in MCF-7 cells (Figures 1C,D).

### 3.2 Effects of “Njansang” oil on weight development

**FIGURE 2:**
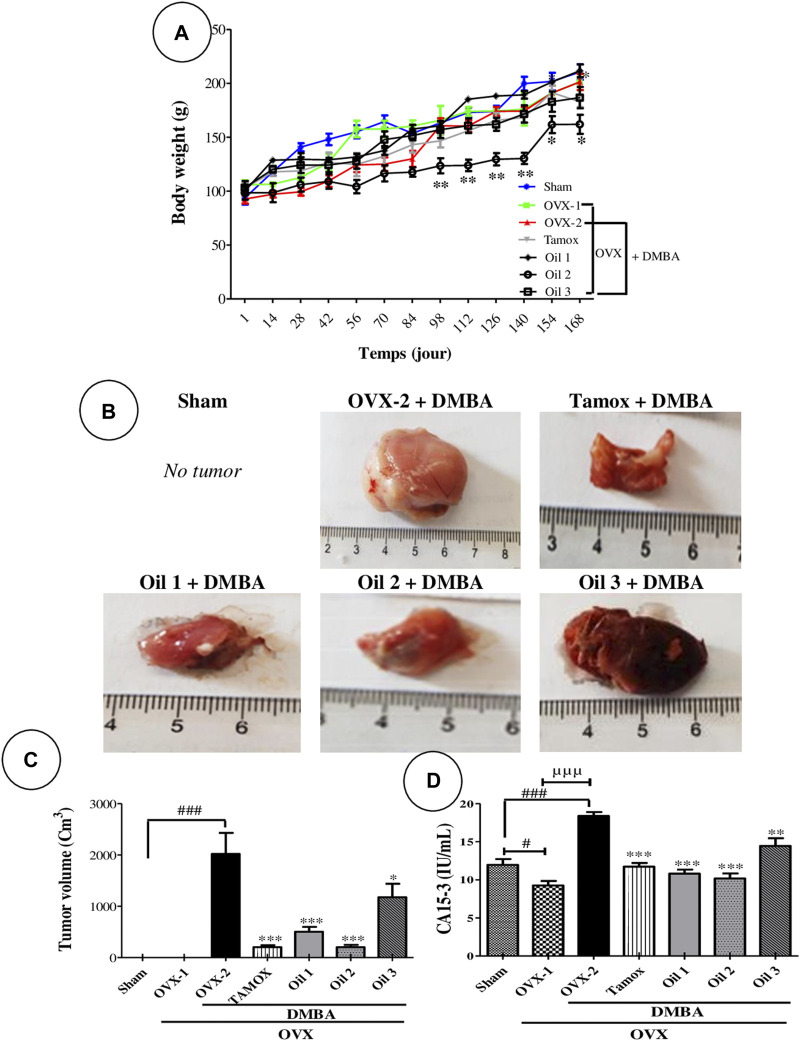
Effects of “Njansang” oil on body weight **(A)**, tumor morphology **(B)**, tumor volume **(C)** and serum CA 15–3 levels **(D)**. Sham operated (Sham), ovariectomized (OVX-1) and negative (OVX-2 + DMBA, DMBA-exposed OVX rats) control groups all received corn oil (vehicle). The Tamox + DMBA group, consisting of positive control rats, received tamoxifen at a dose of 3.3 mg/kg body weight dissolved in corn oil. The test groups consisted of OVX rats treated with “Njansang” oil either 2 times/week (Oil 1), 4 times/week (Oil 2) or daily (Oil 3). Significance compared with the OVX-2 group: **p < 0.01 and ***p < 0.001. Significance compared with the Sham group: #*p <* 0.05 and ###*p <* 0.001. Significance compared with OVX-1 group: ^µµµ^ *p <* 0.001.

Figure 2A shows the evolution of body weight during the 25 weeks of experimentation. Animal growth was normal throughout the study, with no significant changes observed between the OVX and NOR groups on one hand, and OVX-1 and OVX-2 (exposed to DMBA) on the other. However, from day 98 to the end of the experiment, there was a significant drop (at least *p <* 0.05) in the body weight of animals exposed to DMBA and treated with “Njansang” oil daily (Oil 3), compared with the negative control group (OVX-2).

### 3.3 Effects of “Njansang” oil on certain tumor parameters

Table 1 shows that OVX rats exposed to DMBA developed tumors with an incidence of 67% (4 rats/6) compared with the normal (NOR) and ovariectomized (OVX-1) groups. Rats exposed to DMBA and treated with “Njansang” oil twice a week (Oil 1) had 3 rats/6 (50% incidence) with tumors, while rats treated daily (Oil 3) with “Njansang” oil had a tumor incidence of 33.3% (2/6 rats). Only one tumor was recorded in the groups exposed to DMBA and treated with tamoxifen and “Njansang” oil taken 4 times a week (Oil 2).

**TABLE 1:** Chemoprotective activity of “Njansang” oil on breast cancer after 25 weeks of treatment.

Parameters			DMBA				
		OVX					
	Sham	OVX-1	OVX-2	Tamox	Oil 1	Oil 2	Oil 3
Rat with tumor	0/6	0/6	4/6	1/6	3/6	2/6	1/6
Incidence of tumor (%)	0%	0%	67%	17%**	50%*	17%**	33.3%*
Tumor burden (g)	-	-	6.1	1.08**	2.3**	1.08**	3.13**
inhibition of tumor burden	-	-	-	82,78	82,78	65,57*	48,36**
Tumor weight (mg/100 g)	0.38 ± 0.06	0,36 ± 0.02	1.22 ± 0.21	0,21 ± 0.03	0.46 ± 0.03	0.21 ± 0.04	0.63 ± 0.2

Sham operated (Sham), ovariectomized (OVX-1) and negative (OVX-2 + DMBA, DMBA-exposed OVX, rats) control groups all received corn oil (vehicle). The Tamox + DMBA, group, consisting of positive control rats, received tamoxifen at a dose of 3.3 mg/kg body weight dissolved in corn oil. The test groups consisted of OVX, rats treated with “Njansang” oil either 2 times/week (Oil 1), 4 times/week (Oil 2) or daily (Oil 3). Significance compared with OVX-2, group: *p < 0.05 and **p < 0.01.

### 3.4 Effects of “Njansang” oil on tumour volume and CA15-3 levels

Figure 2D shows that CA15-3 levels increased significantly (*p <* 0.001) in the OVX-2 group simultaneously with tumor development, compared with the Sham and OVX groups (Figure 2B). *“*Njansang” oil taken at all frequencies significantly reduced tumor volume and serum CA15-3 levels in the 2- and 4-times-weekly treatment groups (*p <* 0.001). These results were similar to those induced by Tamox (*p <* 0.001) at a dose of 3.3 mg/kg. A decrease in tumor volume and CA15-3 levels was also observed in rats taking “Njansang” oil daily (Oil 3), but at a lower intensity (*p <* 0.01).

### 3.5 Effects of “Njansang” oil on the microarchitecture of mammary glands and mammary tumors

**FIGURE 3:**
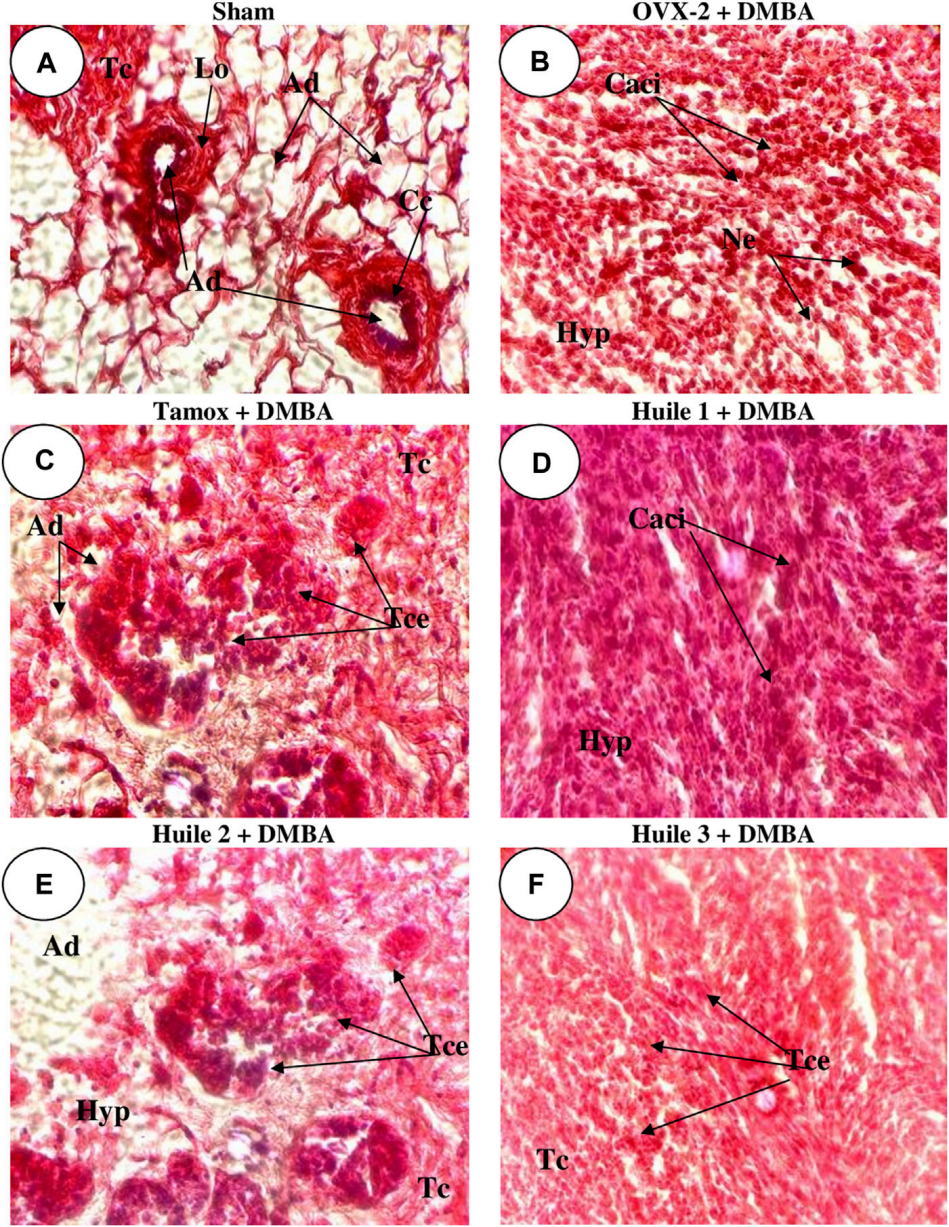
Effects of “Njansang” oil on the microphotographs of mammary glands (and breast tumors). Sham operated [Sham-**(A)**] and negative [OVX-2 + DMBA, DMBA-exposed OVX rats-**(B)**] control groups all received corn oil (vehicle). The Tamox + DMBA group **(C)**, consisting of positive control rats, received tamoxifen at a dose of 3.3 mg/kg body weight dissolved in corn oil. The test groups consisted of OVX rats treated with “Njansang” oil either 2 times/week [Oil 1-**(D)**], 4 times/week [Oil 2-**(E)**] or daily [Oil 3-**(F)**]. Lo = Mammary lobule; Ad = Adipocyte; Cc = Acinar cell layer, Tc = Connective tissue, Hyp = Connective tissue hyperplasia, Caci = Carcinomatous cells, Ne = Necrotic cells, Tce = Cellular trabecula.

Figure 3A shows a mammary gland consisting essentially of normal adipose tissue, dissociated in places by periductal connective tissue arranged around the glandular structures, the lumen of which is surrounded by a double cell bed without atypia (Sham).

Control-negative animals (exposed to DMBA only) present a carcinoma in which the normal architecture of the mammary tissue has been modified and replaced by a tumoral proliferation consisting of trabeculae and rows of carcinomatous cells with various cytonuclear atypia such as anisocytosis, anisokaryosis, mitoses and connective hyperplasia (Figure 3B).

Rats treated with tamoxifen had their normal mammary parenchyma architecture modified and replaced by a tumor proliferation consisting of clusters and rare trabeculae of atypical cells accompanied by conjunctive hyperplasia (Figure 3C).

Rats treated with “Njansang” oil had their mammary gland architecture modified in favor of conjunctive hyperplasia hosting tumor cells arranged in single file and bordering extensive patches of necrosis (Figures 3D–F). The best protection was observed in rats receiving “Njansang” oil four times a week (Oil 2).

### 3.6 Effects of “Njansang” oil on some inflammatory cytokines

**FIGURE 4:**
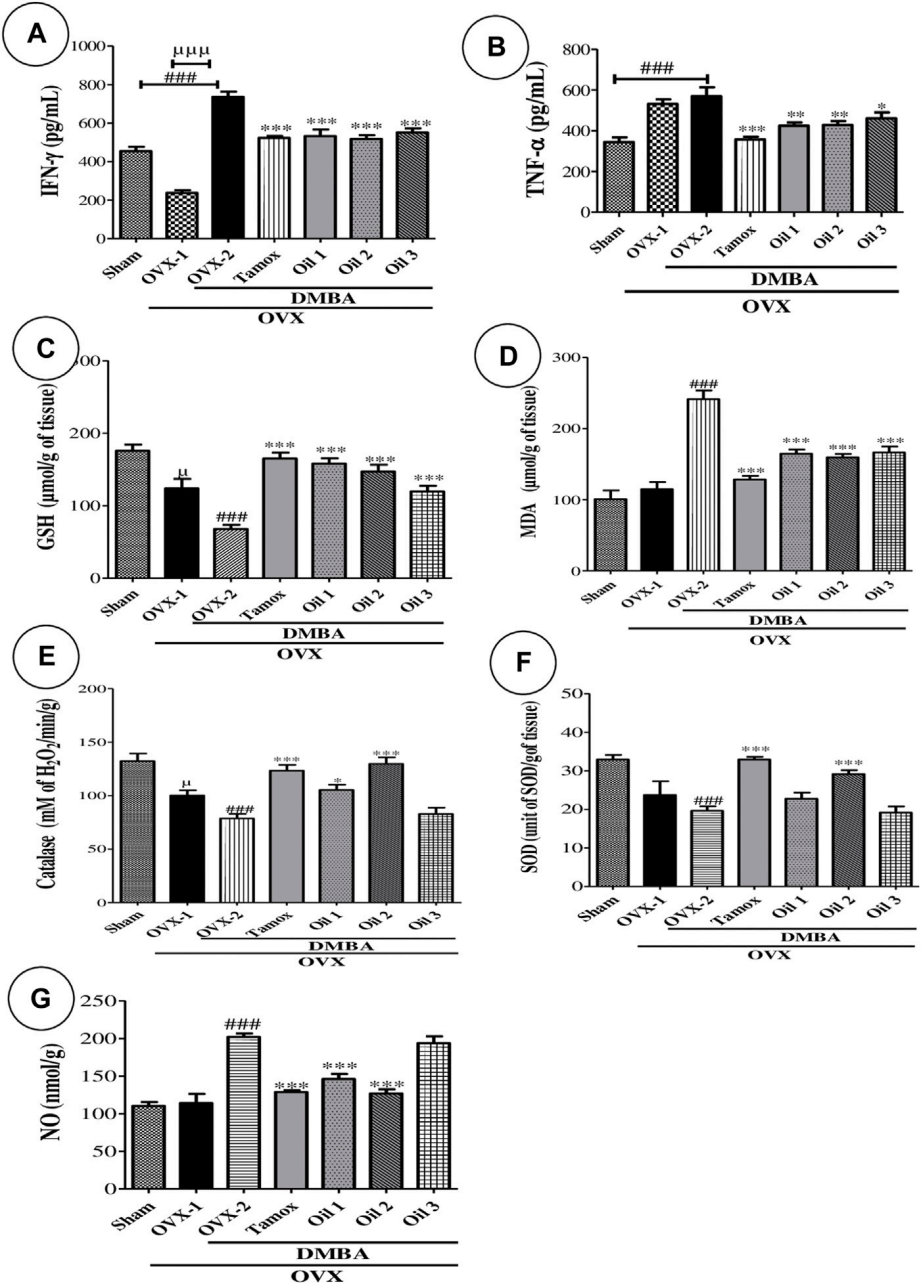
Effects of “Njansang” oil on some inflammatory (INFγ **(A)** and TNF-α **(B)**) and antioxidative parameters (GSH **(C)**, MDA **(D)**, catalase **(E)**, SOD **(F)** and NO **(G)**). Sham operated (Sham), ovariectomized (OVX-1) and negative (OVX-2 + DMBA, DMBA-exposed OVX rats) control groups all received corn oil (vehicle). The Tamox + DMBA group, consisting of positive control rats, received tamoxifen at a dose of 3.3 mg/kg body weight dissolved in corn oil. The test groups consisted of OVX rats treated with “Njansang” oil either 2 times/week (Oil 1), 4 times/week (Oil 2) or daily (Oil 3). Significance compared with OVX-2 group: *p < 0.05, **p < 0.01 and ***p < 0.001. Significance compared with Sham group: ^###^ *p <* 0.001. Significance compared with OVX-1 group: ^µ^ *p <* 0.05 and ^µµµ^ *p <* 0.001.

Figures 4A,B shows the effects of different treatments on the concentrations of some pro-inflammatory cytokines, namely INF-γ (Figure 4A) and TNF-α (Figure 4B). DMBA induced a significant (*p <* 0.001) increase in TNF-α and INF-γ levels. Tamoxifen treatment induced a significant decrease (*p <* 0.001) in TNF-α and INF-γ levels compared with the Sham and OVX-1 groups. All groups treated with “Njansang” oil at all frequencies showed a significant decrease in serum INF-γ (at least *p <* 0.01) and TNF-α (at least *p <* 0.05) compared with the negative control group (OVX-2).

### 3.7 Effects of “Njansang” oil on some parameters of oxidative stress of the mammary glands

Figures 4C–G depicts the effect of “Njansang” oil on certain oxidative stress parameters. MDA levels significantly increased (*p <* 0.001) in rats in the OVX-2 group compared with the NOR and OVX-1 groups. All oil-treated groups at all frequencies showed a significant decrease (*p <* 0.001) in MDA levels compared with the negative controls (OVX-2).

OVX-2 rats showed a significant decrease (*p <* 0.001) in GSH levels compared with the Sham and OVX-1 groups. All oil-treated groups showed a significant increase (*p <* 0.001) in GSH levels compared with the OVX-2 group. Regarding catalase activity, a significant decrease (*p <* 0.001) was observed in rats in the OVX-2 group compared with the Sham and OVX-1 groups. Oil treatments resulted in a significant increase (*p <* 0.01) for Oil 1, (*p <* 0.001) for Oil-2 in catalase activity. SOD activity decreased significantly (*p <* 0.001) in rats in the OVX-2 group compared with the Sham and OVX-1 groups. “Njansang” oil significantly (*p <* 0.001) increased SOD activity in the 4 times/week group, but not significantly in the twice/week and daily groups. Nitric oxide levels in the mammary tumor increased significantly (*p* < 0.001) in the OVX-2 group. Oil treatment significantly decreased (*p <* 0.001) for Oil 1 and 2 the level of NO in the tumor compared to the OVX-2 group.

### 3.8 Effects of “Njansang” oil on the relative mass of some organs and on some hematological parameters

Table 2 shows that DMBA induced a significant decrement in spleen wet weight (*p <* 0.05) compared with the Sham group. Oil taken at full frequency significantly prevented this decrease (*p <* 0.01). A non-significant decrease in white and red blood cell counts in the OVX-2 group compared with normal sham animals was observed. The percentage of monocytes increased significantly (*p* < 0.001) in the OVX-2 group compared with the Sham group. All groups treated with “Njansang” oil at all frequencies showed a significant decrease (*p* < 0.001) in the percentage of monocytes compared with the OVX-2 group. Concerning lymphocytes, oil taken at frequencies of 4 times a week (*p* < 0.05) and daily (*p* < 0.05) resulted in a significant increase in their percentage compared to negative control animals (OVX-2).

**TABLE 2:** Effect of “Njansang” oil on some relative organ weights and hematological parameters.

Organ weight (mg/100 g BW)		OVX					
			DMBA				
	Sham	OVX-1	OVX-2	Tamox	Oil 1	Oil 2	Oil 3
Spleen	0.35 ± 0.013	0.32 ± 0.08	0.17 ± 0.08#00B0F0; >^#^	0.29 ± 0.01#00B0F0; >*	0.35 ± 0.093#00B0F0; >**	0.40 ± 0.03#00B0F0; >**	0.47 ± 0.07#00B0F0; >**
Liver	2.75 ± 0.10	3.23 ± 0.37	3.23 ± 0.37	2.73 ± 0.03	2.73 ± 0.35	2.74 ± 0.25	2.94 ± 0.20
Femur	0.29 ± 0.03	0.36 ± 0.01	0.35 ± 0.01	0.31 ± 0.01	0.30 ± 0.08	0.39 ± 0.03	0.31 ± 0.01
Kidneys	0.51 ± 0.07	0.65 ± 0.04	0.60 ± 0.04	0.53 ± 0.04	0.52 ± 0.04	0.55 ± 0.03	0.06 ± 0.06
Uterus	845.03 ± 195.45	653.21 ± 135.86	655.26 ± 135.86	553.26 ± 24.40	403.67 ± 39.05	405.28 ± 31.92	502.26 ± 39.05
**Hematological parameters**							
GB (×10^3^µL^−1^	10.42 ± 1.34	9.16 ± 0.90	10.17 ± 0.89	11.67 ± 4.47	9.80 ± 1.95	6.78 ± 1.25	13.24 ± 0.90
% lymphocytes	72.92 ± 4.13	61.9 ± 3.25	60.90 ± 3.25	71.80 ± 4.47	70.50 ± 3.40	74.6 ± 30#00B0F0; >*	72.12 ± 2.97#00B0F0; >*
% monocytes	5.03 ± 1.56	34.04 ± 0.40#00B0F0; >^###^	36.74 ± 0.37#00B0F0; >^###^	3.40 ± 1.24#00B0F0; >***	11.88 ± 5.08#00B0F0; >***	12.55 ± 3.96#00B0F0; >***	9.01 ± 5.12#00B0F0; >***
% granulocytes	19.40 ± 1.06	6.05 ± 1.90#00B0F0; >^##^	6.12 ± 1.93#00B0F0; >^##^	20.20 ± 1.81#00B0F0; >**	9.47 ± 3.88	9.24 ± 2.41	18.7 ± 2.61#00B0F0; >**
GB (×10^3^µL^−1^)	10.42 ± 1.34	9.16 ± 0.90	10.17 ± 0.89	11.67 ± 4.47	9.80 ± 1.95	6.78 ± 1.25	13.24 ± 0.90

Sham operated (Sham), ovariectomized (OVX-1) and negative (OVX-2 + DMBA, DMBA-exposed OVX, rats) control groups all received corn oil (vehicle). The Tamox + DMBA, group, consisting of positive control rats, received tamoxifen at a dose of 3.3 mg/kg body weight dissolved in corn oil. The test groups consisted of OVX, rats treated with “Njansang” oil either 2 times/week (Oil 1), 4 times/week (Oil 2) or daily (Oil 3). Significance compared with OVX-2, group: *p < 0.05, **p c 0.01 and ***p < 0.001. Significance compared with Sham: ^#^ *p <* 0.0, ^##^ *p <* 0.01 and, ^###^ *p <* 0.001.

### 3.9 Effects of “Njansang” oil on lipid profile

**FIGURE 5:**
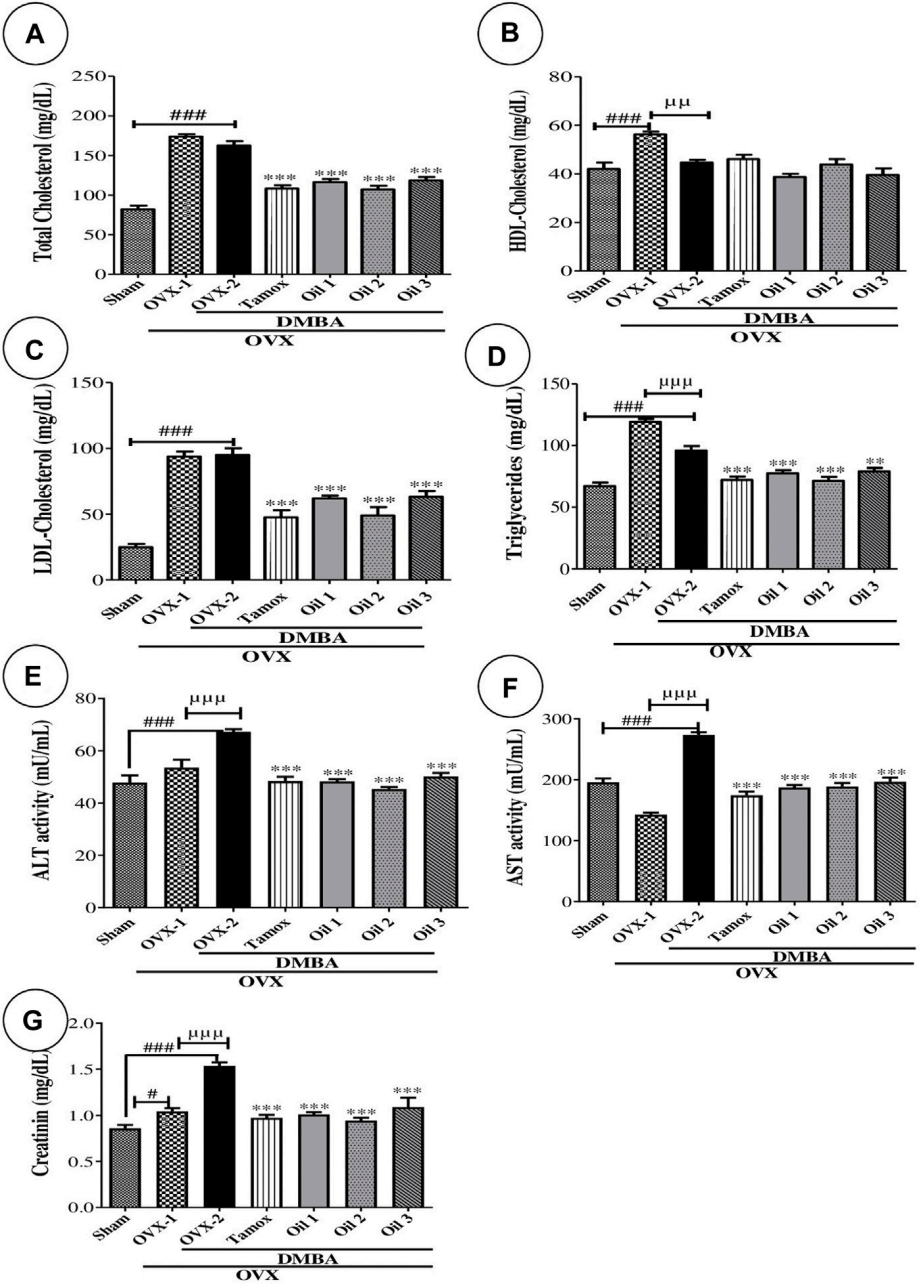
Effects of “Njansang” oil on some lipidemia parameter (Total **(A)**, HDL **(B)**, LDL **(C)** and triglycerides **(D)**) and toxicological parameters (ALT **(E)**, AST **(F)** and creatinine **(G)**). Sham operated (Sham), ovariectomized (OVX-1) and negative (OVX-2 + DMBA, DMBA-exposed OVX rats) control groups all received corn oil (vehicle). The Tamox + DMBA group, consisting of positive control rats, received tamoxifen at a dose of 3.3 mg/kg body weight dissolved in corn oil. The test groups consisted of OVX rats treated with “Njansang” oil either 2 times/week (Oil 1), 4 times/week (Oil 2) or daily (Oil 3). Significance compared with OVX-2 group: **p < 0.01 and ***p < 0.001. Significance compared with Sham group: #*p <* 0.05 and ###*p <* 0.001. Significance compared with OVX-1 group: ^µµ^ *p <* 0.01 and ^µµµ^ *p <* 0.001.

Figure 5 presents the effects of “Njansang” oil on concentrations of total cholesterol (Figure 5A), HDL-cholesterol (Figure 5B), LDL-cholesterol (Figure 5C) and triglycerides (Figure 5D). There was a significant (*p <* 0.001) increase in total, HDL and LDL cholesterol levels in the OVX-2 group compared with the Sham group. The OVX-2 group showed a significant decrease (*p <* 0.01) in HDL-cholesterol levels compared with the OVX-1 group. Compared with OVX-2, all groups treated with “Njansang” oil at all administration frequencies showed significantly (*p <* 0.001) lower levels of total cholesterol, LDL-cholesterol and triglycerides. No significant change was observed with “Njansang” oil in HDL-cholesterol.

### 3.10 Effects of “Njansang” oil on some toxicity parameters

Figures 5E–G shows the effects of different treatments on some markers of liver function (ALT, AST) and kidney function (creatinine). In the OVX-2 group, ALT, AST and creatinine activity increased significantly (*p <* 0.001) compared with the Sham group. Treatment with oil at all dosing frequencies resulted in a significant (*p <* 0.001) decrease in ALT and AST activity and creatinine levels compared to the OVX-2 group, with similar results observed with tamoxifen.

### 3.11 Effects of “Njansang” oil on organ microarchitecture

**FIGURE 6:**
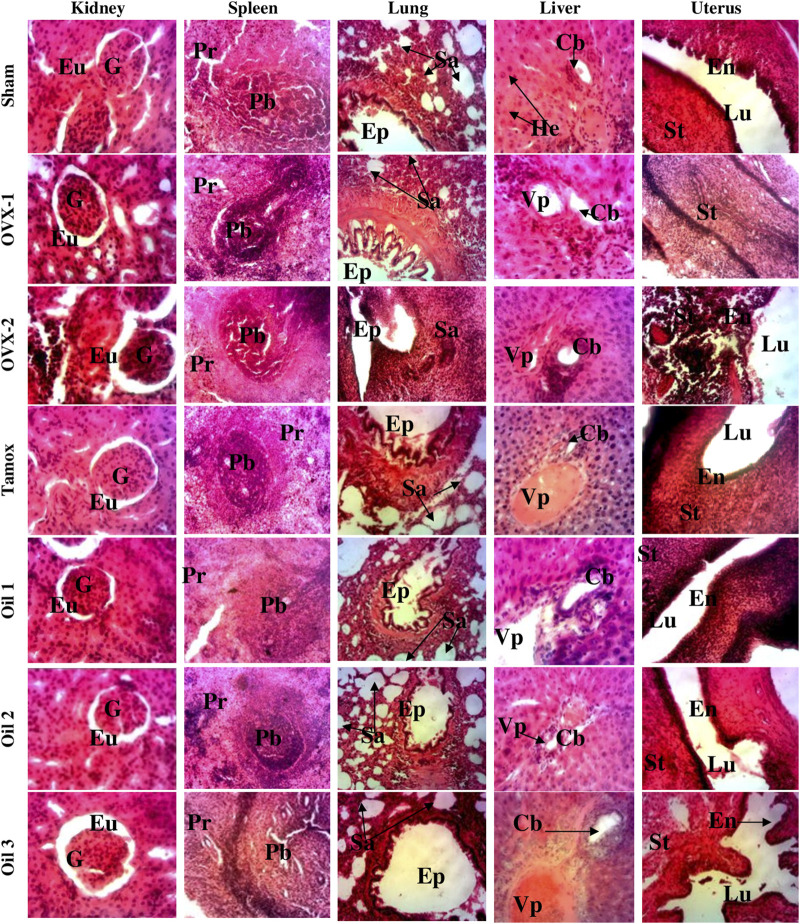
Effects of “Njansang” oil on microarchitechtures of kidneys, spleen, lung, liver and uterus. Sham operated (Sham), ovariectomized (OVX-1) and negative (OVX-2 + DMBA, DMBA-exposed OVX rats) control groups all received corn oil (vehicle). The Tamox + DMBA group, consisting of positive control rats, received tamoxifen at a dose of 3.3 mg/kg body weight dissolved in corn oil. The test groups consisted of OVX rats treated with “Njansang” oil either 2 times/week (Oil 1), 4 times/week (Oil 2) or daily (Oil 3). Go = Glomerulus, Pr = Red pulp, Vp = Portal vein, Lu = Uterine lumen, Eu = Urinary space, G = Glomerulus, Pb = White pulp, Pr = Red pulp, DPb = Disorganization of white pulp, Ep = Pulmonary epithelium, Sa = Alveolar sac, Vp = Portal vein, Cb = Bile duct, M = Medullary layer, S = Interlobular septum, C = Cortex, Lu = Uterine lumen, En = Endometrium, st = Stromal tissue, He = Hepatocytes.

Figure 6 depicts the effects of “Njansang” seed oil on the microarchitecture of the kidney, spleen, liver, lung and uterus after 25 weeks of treatment. Photomicrographs of OVX-2 (DMBA) show leukocyte infiltration in the kidney, liver and lung; disorganization of the white pulp of the spleen compared with normal spleens (Sham). Additionally, it decreased cortical lymphocyte density, showing normal kidney structure with urinary space and well-differentiated glomerulus, well-differentiated white and red pulp in the spleen, normal liver parenchyma with normal hepatocytes and lung with normal alveolar sacs. “Njansang” oil prevented the adverse effects induced by DMBA on these tissues, evidenced by increased cortical lymphocyte density and less leukocyte infiltration in the kidney, liver and lung. Microarchitectures of the spleen were almost normal.

## 4 Discussion

Breast cancer is the most common type of cancer in women worldwide, including Cameroon (Sedeta et al., 2023). Despite the availability of treatments, their drawbacks and high costs limit accessibility, particularly in developing countries where mortality rates remain high due to various systemic issues such as inadequate healthcare infrastructure and convoluted treatment pathways (Geretto et al., 2017; Zingue et al., 2021). Consequently, many patients in these regions turn to traditional medicine, which offers accessibility and minimal side effects. Notably, the World Health Organization reports that 80% of people in Africa and certain Asian countries still rely on herbal remedies for various ailments, including cancer (Regional Committee for Africa, 2020bib_rcfa_2020). Thus, the exploration of natural anti-tumoral substances is imperative, with one significant strategy being the investigation of local natural compounds based on traditional uses (Mbaveng et al., 2017). Numerous research endeavors elucidating tumor transformation mechanisms have underscored the role of diets in cancer prevention (Song et al., 2015).

The cytotoxic activity of *R*. *heudelotii* seed oil was evaluated on MCF-7 cells and MDA-MB 231 cell lines. Cytotoxicity tests are useful for measuring a substance’s ability to induce cell death or inhibit cell growth by altering one or more cellular functions (Weyermann et al., 2005). “Njansang” oil significantly reduced the growth and proliferation rate of MDA-MB 231 cells compared to MCF-7 cells. *Ricinodendron heudelotii* seed has been found to exhibit estrogenic effects probably due to phytoestrogen content (Michel et al., 2022); phytoestrogens have been found to be more active in inhibiting cell growth in MDA-MB-231 breast cancer cells compared to MCF-7 cells. The differential activity may be attributed to the specific signalling pathways and molecular mechanisms involved in each cell line. Additionally, the MDA-MB 231 are triple-negative cancer cells (-ER/- PR/- E-Catherin) in which, the protein have mutated, suggesting that the difference in anti-proliferative efficacy between the tested cell lines could be attributable to one of these pathways. In the case of MDA-MB-231 cells, the growth-inhibiting effects of phytoestrogens may be mediated through the modulation of multiple signalling pathways, including the extracellular matrix (ECM), adhesion, cell cycle, chemokine and cytokine, PI3K/AKT, STAT, TGF-β, MAPK, NF-κB, RAS/Rho, DNA repair, and apoptosis signals (Giordano et al., 2023).

The polycyclic aromatic hydrocarbon (PAH) DMBA is well known to induce breast cancer in female rats undergoing hormonal transition (−60 days), when their terminal mammary ducts undergo active proliferation (Ariazi et al., 2005). In line with the above, 67% of rats exposed to DMBA alone developed mammary tumors in this study. It should be noted that DMBA-induced breast cancer in female rats is a popular model of estrogen-sensitive cancer that develops from ductal epithelial cells and strongly resembles cancer found in women biochemically, immunologically and histologically (Russo and Russo, 2000). In this research, it was observed that “Njansang” oil along with the positive control (tamoxifen), notably decreased the incidence of death and tumors, as well as reducing tumor volume and weight and CA 15–3 levels compared to the OVX-2 and DMBA groups. These findings indicate a protective effect of the oil tested against breast cancer development. The level of the biomarker CA 15–3 was significantly increased in the serum of rats in the OVX-2 group. CA 15–3 is an important prognostic factor in breast cancer and is generally associated with a high rate of cell proliferation (Duffy et al., 2010).

Inflammation plays an important role in tumor initiation, promotion, angiogenesis and metastasis (Murugaiyan and Saha, 2009). Serum levels of TNF-α (an inflammatory agent highly expressed in breast carcinomas) increased following DMBA exposure (Ahmad et al., 2017; Kalyani et al., 2017). Pro-inflammatory mediators such as TNF-α and IL-6 are key players in cancer-related inflammation, and inhibition of these cytokines could protect against chemically induced breast tumors (Kalyani et al., 2017). DMBA induced an increase in TNF-α levels compared to NOR and OVX-1 rats. Oil extracted from *R. heudelotii* seeds significantly (*p <* 0.001) reduced serum TNF-α levels compared with the OVX-2 group. This reduction is thought to inhibit tumor proliferation and prevent the secretion of several pro-inflammatory cytokines such as IL-17 and IL-6 (not measured in this study). TNF-α is also a tumor-promoting necrosis factor in the tumor microenvironment, highly expressed in breast adenocarcinomas. A decrease in these inflammatory cytokines suggests that *R. heudelotii* oil is rich in anti-inflammatory compounds. Elevated serum INF-γ is generally correlated with tumor growth, and its reduction is a good prognostic in cancer management (Ni and Lu, 2018). It increased significantly (*p <* 0.001) in this study. *R. heudelotii* oil significantly (*p <* 0.001) reduced INF-γ levels compared with the OVX-2 group hence the anti-cancer effect of Njansang oil.

To better understand the protective effect of *R. heudelotii* oil, the antioxidant status of the animals was assessed. Indeed, it is well established that DMBA-induced mammary carcinoma in rats acts in part through the production of reactive oxygen species (ROS), which in turn damage DNA (Kalyani et al., 2017). According to Rašić (2018), serum MDA levels are extremely higher in cancer patients than in normal patients, consistent with the significant (*p* < 0.001) increase in MDA levels observed in the OVX-2 group compared with the NOR and OVX-1 groups. Rats treated with *R. heudelotii* oil at all frequencies just like tamoxifen were significantly (*p* < 0.001) protected against increased MDA levels, suggesting protection from cell membrane damage. An increase in SOD and catalase activities as well as GSH levels and a decrease in MDA levels compared to the OVX-2 group were noted. It should be noted that SOD and catalase are ubiquitous cytoplasmic enzymes that protect cells from free radicals produced during carcinogenic metabolism (Kalyani et al., 2017). Their significant increase (*p* < 0.001) was noted in the group taking “Njansang” oil 4 times/week (*p* < 0.05), suggesting its ability to scavenge free radicals, which is in agreement with the *in vivo* results. In addition, GSH, which is one of the first lines of defense against reactive oxygen species, was increased and MDA (a marker of lipid peroxidation) was decreased compared to the negative control group. These results confirm the antioxidant effect of “Njansang” oil, which in turn may explain its anti-cancer effects. Nitrites are one of the precursors of ROS and are also a critical risk factor for breast cancer (Druckrey et al., 1967). In this study, during active inflammation of breast cancer, higher concentrations of nitrites were observed in mammary tissue compared to normal rats. “Njansang” oil significantly decreased (*p* < 0.001), at all frequencies tested except for those taking “Njansang” oil daily, nitric oxide levels in mammary tissue compared with the OVX-2 control group. This suggests that Njansang oil exerts some of its anti-cancer effects through antioxidant properties.

Changes in relative organ weights are an indicator of the potential side effects of toxic substances (Piao et al., 2013). DMBA induced a significant increase in the weight of the spleen, uterus and mammary gland, compared with NOR and OVX-1 rats. This would be due to the immunotoxicity of DMBA (Miyata et al., 2001); however, all treated groups showed a significant decrease (*p* < 0.001) compared to the OVX-2 group. Indeed, DMBA induced an increase in fresh spleen weight, which could signify anemia (Silihe et al., 2017). The decrease in fresh liver weight does not corroborate with the increase in ALT activity, which shows that there is no hepatocyte destruction. The kidney is an important organ responsible for the excretion of various toxic metabolic wastes. It cannot escape the harmful effect of DMBA’s toxic metabolic products. In the present study, although no significant change was noted in kidney weight, DMBA significantly (*p* < 0.001) increased serum creatinine levels in the OVX-2 group compared with the NOR and OVX-1 groups; indicating kidney damage (Dakrory et al., 2015). “Njansang” oil significantly (*p* < 0.001) decreased serum creatinine levels compared to the OVX-2 group in this study. This confirms its non-toxic status as well as its hepato- and protective effects. The kidney is an important organ responsible for the excretion of various toxic metabolic wastes. It cannot escape the harmful effect of DMBA’s toxic metabolic products. In the present study, although no significant change was noted in kidney weight, DMBA significantly (*p* < 0.001) increased serum creatinine levels in the OVX-2 group compared with the NOR and OVX-1 groups; indicating kidney damage (Dakrory et al., 2015). “Njansang” oil significantly (*p* < 0.001) decreased serum creatinine levels compared to the OVX-2 group in this study. This confirms its non-toxic status as well as its hepato- and renal-protective effects.

## 5 Conclusion

*In vitro,* “Njansang” oil significantly reduced the growth of ER+ (MCF-7) and triple negative (MDA-MB 231) adenocarcinoma cells. *In vivo*, 67% of DMBA-exposed rats developed at least one tumor. Both “Njansang” oil and the standard treatment (tamoxifen) significantly reduced tumor incidence, tumor weight, tumor histopathology and serum CA 15–3 levels compared to diseased animals. “Njansang” oil exhibited antioxidant activity, as evidenced by increased SOD and catalase activities and elevated GSH levels, while decreasing MDA levels compared to OVX-2. It also significantly reduced inflammatory mediators such as TNF-α and INF-γ levels, as well as serum levels of total cholesterol, LDL-cholesterol and triglycerides. Taken together, these results confirm the antioxidant, lipid-lowering and anti-inflammatory actions of *R*. *heudelotii* through which it may exert its beneficial effects on cancer. In the future, we propose to characterize the bioactive compounds present in “Njansang” oil and evaluate the in-depth toxicity of “Njansang” oil.

## Data Availability

The raw data supporting the conclusion of this article will be made available by the authors, without undue reservation.

## Abbreviations

WHO, World Health Organization; PAHs, polycyclic aromatic hydrocarbons DMBA, 7,12-dimethylbenz(a)anthracene; E2V, 17β-estradiol Valerate; MDA-MB 231, Human estrogen non-sensitive; MCF-7, Human estrogen sensitive; ATCC, American Type Culture Collection; FBS, Fetal bovine serum; MTT, Mitochondrial tetrazolium test; ELISA, Enzyme-linked immunosorbent assay; CCK-8, Cell Counting Kit-8; SERM, Selective estrogen receptor modulator; EDTA, Ethylene diamine tetra-acetic; Hematocrit (Htc); Hb, Hemoglobin; MCV, Mean corpuscular volume; MCHC, Mean corpuscular hemoglobin concentration; MUC-1, Mucin-1 protein; SEM, Standard error of the mean; ANOVA, Analysis of variance.
